# A critical role for ecdysone response genes in regulating egg production in adult female *Rhodnius prolixus*

**DOI:** 10.1371/journal.pone.0283286

**Published:** 2023-03-20

**Authors:** Samiha Benrabaa, Ian Orchard, Angela B. Lange

**Affiliations:** Department of Biology, University of Toronto Mississauga, Mississauga, ON, Canada; Universidade Federal do Rio de Janeiro, BRAZIL

## Abstract

Ecdysteroids control ovary growth and egg production through a complex gene hierarchy. In the female *Rhodnius prolixus*, a blood-gorging triatomine and the vector of Chagas disease, we have identified the ecdysone response genes in the ovary using transcriptomic data. We then quantified the expression of the ecdysone response gene transcripts (*E75*, *E74*, *BR-C*, *HR3*, *HR4*, and *FTZ-F1*) in several tissues, including the ovary, following a blood meal. These results confirm the presence of these transcripts in several tissues in *R*. *prolixus* and show that the ecdysone response genes in the ovary are mostly upregulated during the first three days post blood meal (PBM). Knockdown of *E75*, *E74*, or *FTZ-F1* transcripts using RNA interference (RNAi) was used to understand the role of the ecdysone response genes in vitellogenesis and egg production. Knockdown significantly decreases the expression of the transcripts for the ecdysone receptor and Halloween genes in the fat body and the ovaries and reduces the titer of ecdysteroid in the hemolymph. Knockdown of each of these transcription factors typically alters the expression of the other transcription factors. Knockdown also significantly decreases the expression of vitellogenin transcripts, *Vg1* and *Vg2*, in the fat body and ovaries and reduces the number of eggs produced and laid. Some of the laid eggs have an irregular shape and smaller volume, and their hatching rate is decreased. Knockdown also influences the expression of the chorion gene transcripts *Rp30* and *Rp45*. The overall effect of knockdown is a decrease in number of eggs produced and a severe reduction in number of eggs laid and their hatching rate. Clearly, ecdysteroids and ecdysone response genes play a significant role in reproduction in *R*. *prolixus*.

## Introduction

Ecdysteroids play a crucial role in insect development, metamorphosis, and reproduction [1, 2] Ecdysteroids include ecdysone (E), produced from cholesterol, and 20-hydroxyecdysone (20E), the biologically active hormone, produced from the conversion of E by cytochrome P450 enzymes. 20E binds to a heterodimeric nuclear hormone receptor consisting of the ecdysone receptor (homolog of the mammalian farnesoid X receptor) and utraspiracle (homolog of the mammalian RXR) (EcR /USP). The ecdysone receptor complex acts as a transcription factor, by binding to an enhancer region known as the ecdysone response element (EcRE). This enhancer region is located upstream of the regulatory region of genes known as the ecdysone response genes [3–8]. *E75*, *E74*, and *BR-C* are early ecdysone response genes induced by the ecdysone receptor complex followed by an upregulation of early-late genes *HR3*, *HR4* in conjunction with 20E-induced protein synthesis. *FTZ-F1* is a downstream gene in the pathway of 20E and has also been reported to be a 20E-responsive gene in the prothoracic gland [8–10]. These then regulate the expression of late ecdysone response genes directing a particular biological response to 20E [11, 12]. In this model, the 20E/receptor complex serves two functions; directly activating early genes and repressing late genes. The proteins encoded by the early genes also exert two regulatory functions; activating late genes and suppressing their own expression [1, 11, 13]. The transcription factors *BR-C*, *E74* and *E75* encode DNA binding proteins, with *BR-C* and *E75* proteins containing zinc fingers, while *E74* encodes proteins with a complete main domain (ETS) [9, 11, 14]. *E75*, *E74* and *BR-C* bind to early and late genes, revealing their central role in gene regulation [15–17]. It has been demonstrated that mutations in the *E74*, *BR-C* and *E75* early ecdysone response genes are associated with perturbed transcription of other genes within the regulatory hierarchy and reduced ecdysone titer [11, 18, 19]. RNAi experiments that silence *FTZ-F1* and *HR3* genes disrupt normal insect development and metamorphosis [20–25].

Interestingly, an interplay among these 20E-responsive genes has been also reported. *βFTZ*-*F1* is a competence factor necessary for *BR-C*, *E74A*, *E75A* expression after the ecdysteroid peak that induces the prepupa-to-pupa transition [25]. At the same time, *βFTZ*-*F1* is part of the dynamic feedback regulation of 20E-EcR signaling, enhancing ecdysteroid biosynthesis in the prothoracic gland and probably in the ovaries [8]. The role of 20E in reproduction has been reported in both holometabolous and hemimetabolous insects, including *Bombyx mori*, *Aedes aegypti*, *Drosophila melanogaster*, *Nilaparvata lugens*, and *Schistocerca gregaria*, where 20E is involved in aspects of oogenesis [26–29]. Ecdysone response genes are required for vitellogenesis, with ecdysone receptor binding sites and transcriptional regulators *E74* and *E75* identified in the 5′ upstream regulatory region of the vitellogenin (*Vg*) gene [30, 31]. Moreover, the *BR-C* gene is essential for choriogenesis and endoreplication in *D*. *melanogaster* [32].

In adult *R*. *prolixus* females, a blood meal provides the nutrients necessary for full egg production [33]. Ecdysteroids have been reported to initiate ovulation and egg-laying, whereby hemolymph ecdysteroids and a mating factor stimulate medial neurosecretory cells to release a myotropin. The myotropin stimulates contraction of the ovaries, leading to ovulation and egg-laying [33–35]. Ecdysteroid titer peaks in the hemolymph five days PBM in both mated and virgin females, and ovariectomy reduces the ecdysteroid titer. Thus, ecdysteroid is synthesized and released from the ovaries [34, 36, 37]. Recently we identified the Halloween genes and the ecdysone receptor in the ovary of *R*. *prolixus* [37]. Knockdown of *EcR*, *USP* or *shade* transcripts significantly and dramatically reduced the number of eggs made and laid, and reduced the hatching rate. Also, knockdown of *EcR*, *USP* or *shade* reduced the transcript expression of the vitellogenin genes *Vg1*and *Vg2* in the fat body, and significantly reduced the number of developing oocytes. Hence, the ecdysteroid signaling pathway is involved in vitellogenin synthesis in the fat body [37]. In addition, knockdown of the *EcR*, *USP* or *shade* transcripts alters the expression of the chorion gene transcripts *Rp30* and *Rp45*, which affected the shape of the eggs laid [37].

Currently, only one ortholog for each of the ecdysone response genes, *E75*, *HR3*, *HR4*, and *FTZ*-*F1*, has been reported to be present in the *R*. *prolixus* genome [38]. It must be born in mind that this is not the case in other insects. For example, four *E75* mRNA isoforms are found in *D*. *melanogaster*, *E75A*, *E75B*, *E75C*, and *E75D*. In the holometabolous species M*anduca sexta*, the same number of isoforms of *E75* have been described, while *B*. *mori* and *A*. *aegypti* have only three, A, B and C, and in the hemimetabolous species *Blatella germanica*, five isoforms are present [11, 39–41]. Additionally, *E74*, *HR3*, and *BR*-*C* have different mRNA isoforms and different expression patterns in insects; for example, there are two isoforms of the *E74* gene in *D*. *melanogaster* and *A*. *aegypti*, and three isoforms of *HR3* in *B*. *germanica*, named *HR3*-*A*, *HR3*-*B1* and *HR3*-*B2* [21]. Also, in *D*. *melanogaster*, several *BRC* isoforms are produced by alternative splicing among duplicated exons [9, 17]. While there are two isoforms of *FTZ*-*F1* in *D*. *melanogaster*, which are produced by alternative transcription and splicing [25, 42], some insect species have only documented one transcript of *FTZ*-*F1* [43, 44], including *R*. *prolixus* [38].

The role of the individual participants in the hierarchy of ecdysteroid signaling, however, is still largely unknown during reproduction in female *R*. *prolixus*. In this paper, we identify and characterize the ecdysone response genes *E75*, *E74*, *BR-C*, *HR3*, *HR4*, and *FTZ-F1* in *R*. *prolixus* and demonstrate that these genes are essential for normal egg production. We hypothesized that ecdysone response genes are required in 20E biosynthesis and oocyte maturation in the female ovary of *R*. *prolixus*.

## Materials and methods

### Insects

*R*. *prolixus* were taken from a colony kept at 25°C and ~50% relative humidity and fed on defibrinated rabbit blood (Cedarlane Laboratories Inc., Burlington, ON, Canada). The experimental adult insects were previously blood fed as 5^th^ instars. Newly ecdysed adult females and males were separated and maintained at 28°C with a 12 h light:12 h dark regime. Some experiments used mated adult females which had been fed at 10 days post ecdysis (PE) and only insects feeding 2.5–3 times their body weight was used.

### Identification of gene sequences in the ovary transcriptome

An analysis of VectorBase was conducted to extract the sequences of the *R*. *prolixus* ecdysone response genes (*E75*, *E74*, *BR-C*, *HR3*, *HR4*, and *FTZ-F1*). ExPASy2 tool (www.expasy.org) was used to deduce amino acid sequences and a tBLASTn web portal (https://blast.ncbi.nlm.nih.gov/Blast.cgi) was used to run BLAST against the VectorBase genes. The high-level contigs matching with NCBI were confirmed by the e-value and amino acid sequence identity of the top contig hits. The ecdysone response genes were extracted from VectorBase with *E74*, *E75*, *BR-C*, and *HR4* having one contig each. There are two contigs for *FTZ*-*F1* (RPRC001915 and RPRC002968) with the top hit in BLAST for both being *FTZ*-*F1β*. Using Conserved Domain Database (CDD) software (https://www.ncbi.nlm.nih.gov/Structure/cdd/wrpsb.cgi) to analyze the main domain, it was found that RPRC002968 has both domains, the ligand-binding domain (LBD) and DNA-binding domain (DBD), while RPRC001915 is fragmented and has only the LBD domain. We therefore used RPRC002968 in this study. *HR3* has two contigs, RPRC003681 and RPRC000824. While RPRC003681 has a complete DBD with N-terminus, it lacks the LBD while another contig RPRC000824 has only an LBD domain, suggesting that these are fragments of the same gene. The CDD analysis showed that *E74* has an ETS with an N- and C-terminal. In addition, *E75* and *HR4* have DBD and LBD with an N- and C-terminal. BR-C sequences have complete main domain BTB (Broad-Complex, Tramtrack and Bric à brac). After confirming the extracted contig of the genes, their transcript expression in an ovary transcriptome was verified [45]. Phylogenetic trees were generated based on the sequences of the *R*. *prolixus* genes and other arthropod sequences using the MEGA Software (https://www.megasoftware.net). Evolutionary history was estimated using a maximum likelihood and a matrix-based model based on JTT [46]. Based on a matrix of pairwise distances estimated using the JTT model, the Neighbor-Joining and BioNJ algorithms were applied to an initial tree and the topology with the highest log likelihood value was selected. Thirty protein sequences were analyzed. The evolutionary analyses were conducted in MEGAX [47].

### RNA extraction and reverse transcription/quantitative PCR (RT-qPCR)

Adult female *R*. *prolixus* tissues were dissected in cold autoclaved phosphate-buffered saline (PBS, 8.2 mM Na_2_HPO_4_, 1.5 mM KH_2_PO_4_, 150 mM NaCl, 2.7 mM KCl). Total RNA was extracted according to the manufacturer’s instructions using the TRIzol reagent (Invitrogen by ThermoFisher Scientific, MA, USA), and the final concentration and A260/280 ratio of purified RNA determined using a spectrophotometer DS-11+ (DeNovix Inc., Wilmington, DE, USA). To treat total RNA extracts with DNase, a DNase I (RNase-free) Kit (ThermoFisher Scientific Inc., Mississauga, ON, Canada) was used, according to the manufacturer’s instructions. Reverse transcriptase (High-Capacity cDNA Reverse Transcription Kit, Applied-Biosystems, by Fisher Scientific, Mississauga, ON, Canada) was used to synthesize cDNA from 1 μg of total RNA. Random primers and 50 units of MultiScribe MuLV reverse transcriptase were used in accordance with the manufacturer’s instructions. Quantitative PCR (qPCR) was performed as previously described [37], using 4 pmol of forward and reverse primers added to a Supergreen Low Rox Reagent (Wisent Bioproducts Inc, Saint-Jean-Baptiste, QC, Canada) and Advanced Master Mix (S1 Table). Although in *R*. *prolixus* only one ortholog for each of the ecdysone response genes has been reported, we cannot rule out isoforms, and so this presents a limitation on whether the qPCR is detecting only one isoform or different isoforms. The target gene expression was normalized using *actin*, *Rp49* (*60S* ribosomal protein L32) or *18S* ribosomal RNA (*18S* rRNA) as housekeeping genes. The expression of mRNA was calculated relative to 1000 copies of the average of the reference genes with the 2^−ΔCt^ method, while fold change was quantified relative to the expression of control samples, using the 2^−ΔΔCt^ method and the geometric mean of the reference genes [48, 49].

### Ecdysteroid ELISA

The 20E titer was measured in fed female adult insects at 4 and 5 days PBM. Hemolymph was collected using a Hamilton syringe (Hamilton Company, Reno, NV, USA), mixed with 100% methanol at a ratio of 1:3, and stored at -20°C. The hemolymph 20E titers were quantified by competitive ELISA [37, 50].

### Double-stranded RNA design and synthesis

The *E75*, *E74*, and *FTZ-F1* transcripts were downregulated using double-stranded RNAs (dsRNA). For *E75*, dsRNA primers cover LBD (conserved domain) and part of the C terminal, a less conserved region; for *E74*, primers cover part of the unique N-terminal region and the main conserved domain ETS domain; and for *FTZ*-*F1*, primers cover the N-terminal A/B domains. In general, 20E-responsive genes isoforms differ in the N-terminal. Using T7 RNA polymerase, PCR was used to synthesize non-overlapping fragments of the target gene. The dsRNA sequences were compared with the *R*. *prolixus* genome in VectorBase using BLAST. One high confidence hit was found for each dsRNA confirming their specificity. dsRNA was synthesized using the T7 Ribomax Express RNAi System (Promega, Madison, WI, USA). dsARG (Ampicillin Resistance Gene) from the pGEM-T Easy Vector system (Promega, Madison, WI, USA) was used as control.

### Knockdown of transcript expression using double-stranded RNA

Adult females were divided after ecdysis into four groups for injection of dsE75, dsE74, dsFTZ-F1, or dsARG. Insects were injected with 5 μg of dsFTZ-F1 (1 μg/μL) on day 3 PE, or with 5 μg of dsE75 (1 μg/μL) or 1.25 μg of dsE74 (0.25 μg/μL) on day 7 PE. The controls were injected with 2.5 μg of dsARG (0.5 μg/μL) on the equivalent days as the experimental groups.

Significant knockdown of the transcripts was observed at these doses and days (S1 Fig). Following a blood meal on day 10 PE, all females were kept in cubicles with two males at 28°C and 50% humidity. The cubicles were examined for the ejected spermatophores to verify mating. On day 4 PBM, the expression of the *E75*, *E74*, and *FTZ-F1* transcripts in an individual ovary or fat body was determined as described above. RT-qPCR was also used to determine the levels of vitellogenin transcripts, *Vg1* and *Vg2*, in the ovaries and fat body. A minimum of four independent biological samples were analyzed at each time point post injection. The ovaries of dsRNA-injected female *R*. *prolixus* were dissected and photographed at 4 days PBM using a Leica DVM6 digital microscope (Leica Microsystems, Wetzlar, Germany).

### Egg-laying assay

Mated and fed females begin to lay eggs 5–6 days after a blood meal, and egg-laying typically occurs over 20–30 days post feeding [45]. The eggs laid by dsRNA-injected insects (as described above) were counted for 15 days PBM. Eggs were photographed using a Leica DVM6 digital microscope (Leica Microsystems, Wetzlar, Germany). To measure the volume of the eggs, 15 eggs were randomly selected from each dsRNA group and volume was calculated using the equation 1/6 π(length)(width)^2^ [51]. The hatching rate was also determined.

### Statistical analyses

Results are expressed as mean ± SEM. Graphs were created using GraphPad Prism 7 (GraphPad Software, San Diego, California, USA). All data sets passed the normality and homoscedasticity tests. For experiments with multiple groups, one-way ANOVA and Tukey’s post-hoc test was used, while Student’s t-tests were used for comparison between two values. The statistical significance of each case was determined by a P value of < 0.05.

## Results

### Identification of ecdysone response genes in the ovary transcriptome of *R*. *prolixus*

**Fig 1:**
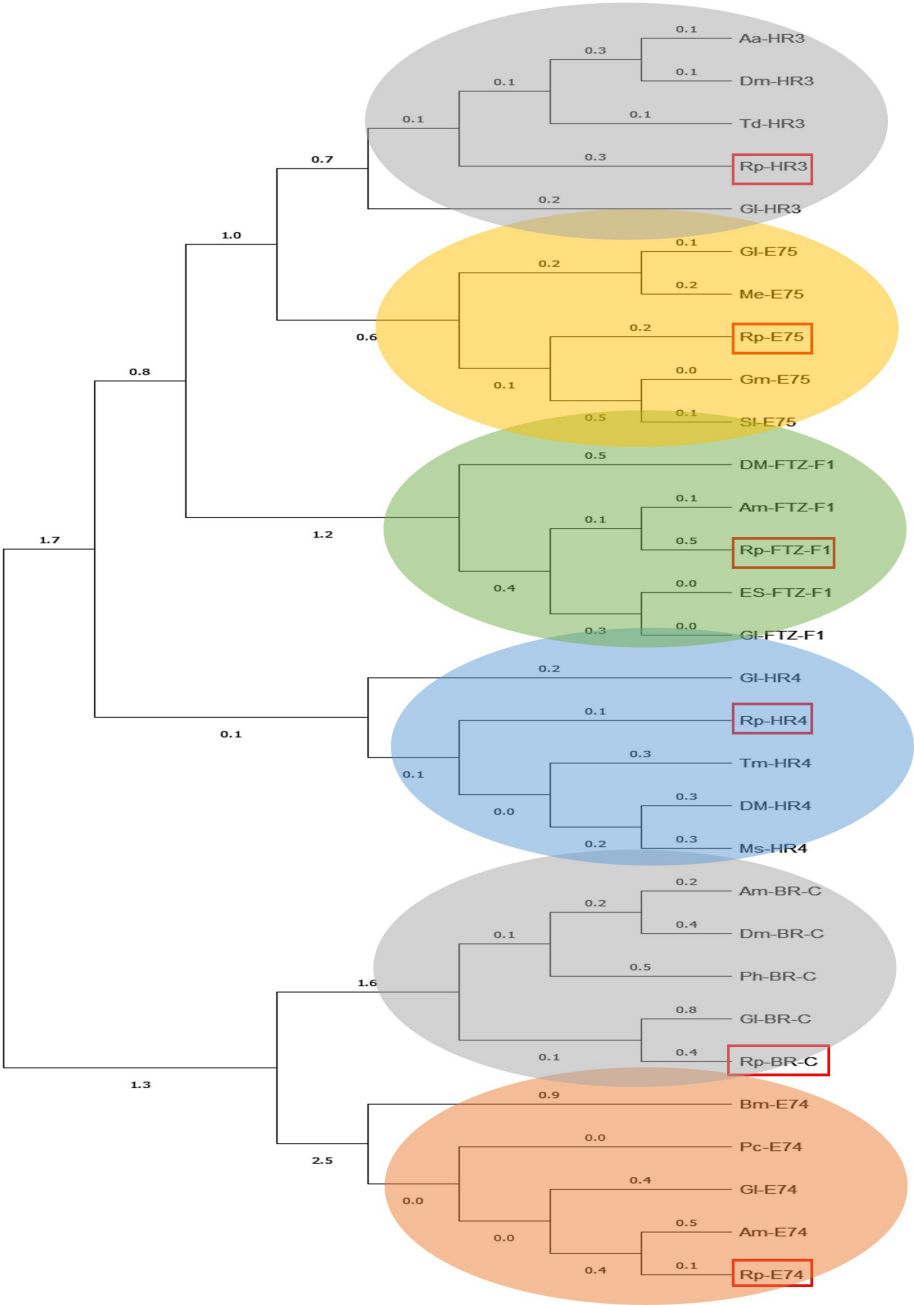
Phylogenetic trees showing the relationship between protein sequences of ecdysone-response genes of *R*. *prolixus* and other arthropod species. Abbreviations: Aa (*Aedes aegypti*), Dm (*Drosophila melanogaster*), Td (*Thermobia domestica*), Gl (*Gecarcinus lateralis*), Me, (*Metapenaeus ensis*), Gm (*Galleria mellonella*), Sl (*Spodoptera littoralis*), Am (*Apis mellifera*), Es (*Eriocheir sinensis*), Tm (*Tenebrio molitor)*, Bm (*Bombyx mori*), Pc (*Phaedon cochleariae*), Pc (*Psacothea hilaris*).

Ecdysone response genes *E75*, *E74*, *BR-C*, *HR3*, *HR4*, and *FTZ-F1* were identified using insect orthologs in the GenBank database. The *R*. *prolixus* sequences were verified by BLAST against the GenBank database. The *R*. *prolixus* genes are all full-length except for *HR3* and *HR4*, which is a fragment (S2 Table). MEGAX software was used to generate a phylogenetic tree. The phylogenetic tree illustrates that the ecdysone response genes of *R*. *prolixus* cluster with their orthologous sequences from other arthropod species (Fig 1). All of the ecdysone response genes are expressed in the ovary transcriptome (S2 Table).

### Tissue transcript expression of ecdysone response genes

**Fig 2:**
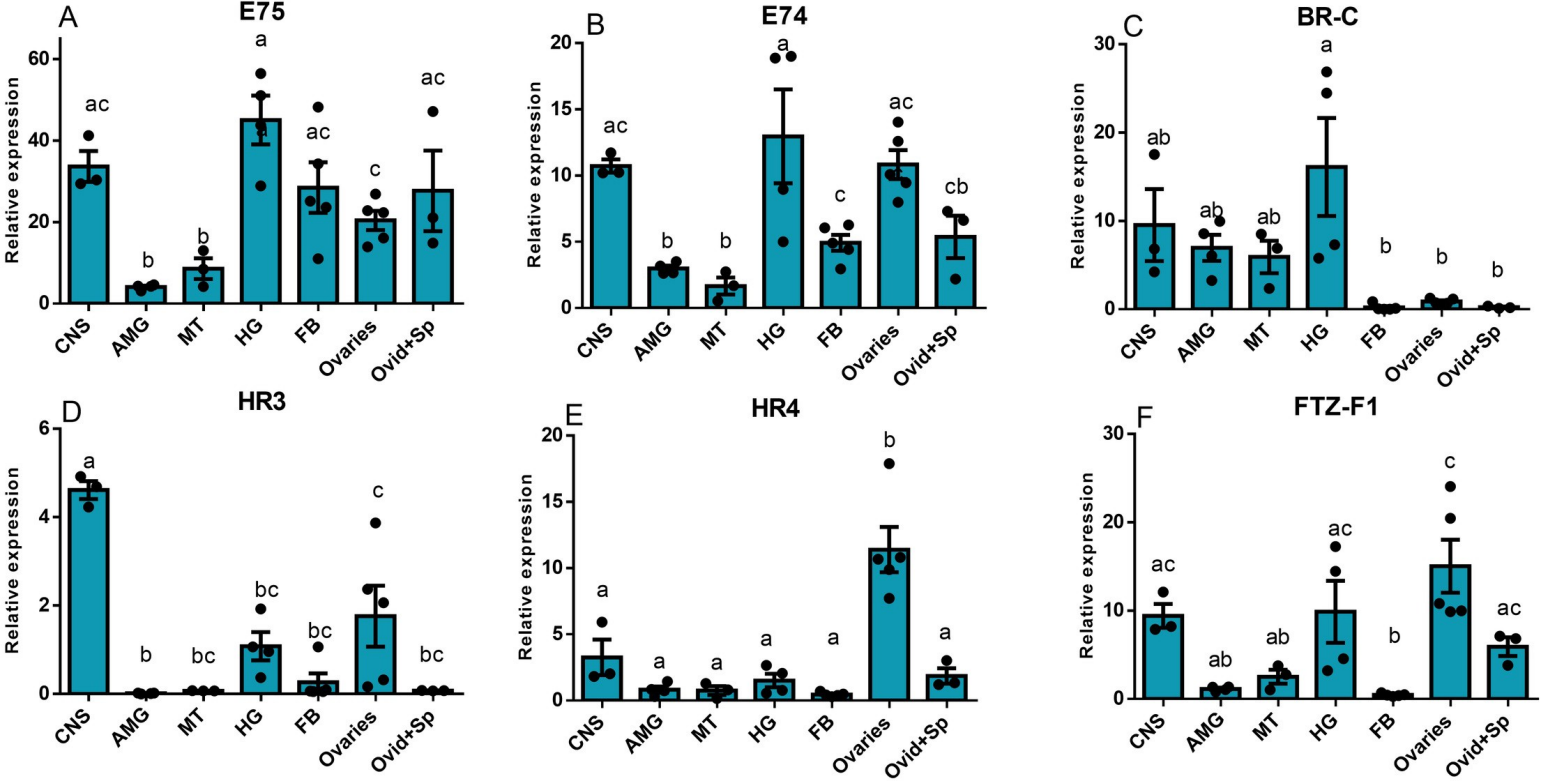
Ecdysone response genes are expressed in the ovary of *R*. *prolixus* adult females. Tissues from three unfed females of *R*. *prolixus* were pooled and separated into the following groups: central nervous system (CNS), anterior midgut (AMG), Malpighian tubules (MT), hindgut (HG), fat body (FB), ovaries (OV), and oviducts (OviD) + spermatheca (Sp). RT-qPCR was used to quantify the transcript expression of *E75* (**A**), *E74* (**B**), *BR-C* (**C**), *HR3* (**D**), *HR4* (**E**), and *FTZ-F1* (**F**). The transcript levels were quantified using RT-qPCR and analyzed using the 2^−ΔCt^ method using *18S rRNA* and *β-actin* as reference genes. The results are shown as the mean ± SEM (n = 3–5). Statistically significant differences at p < 0.05 are indicated by different letters (One-way ANOVA and Tukey’s post hoc test).

Transcript levels of the ecdysone response genes (*E75*, *E74*, *BR-C*, *HR3*, *HR4*, and *FTZ-F1*) were quantified from adult female tissues at 10 d PE from unfed insects using qPCR. Eight tissues were examined: central nervous system (CNS), anterior midgut (AMG), Malpighian tubules (MTs), hindgut (HG), fat body (FB), ovaries (OV), and oviduct (Ovid) + spermatheca (Sp). Transcript expression varies among the tissues collected (Fig 2A–2F), but overall, *E75* is the most highly expressed, and *HR3* is the lowest expressed transcript. With regard to the reproductive tissue, *E75*, *E74*, *HR3*, *HR4*, and *FTZ-F1* transcripts are all expressed in the ovaries, but *BR-C* mRNA transcript expression is relatively low (Fig 2A–2F). In addition, *E75*, *E74*, *HR4*, and *FTZ-F1* transcripts are expressed in the oviducts and spermatheca, along with much lower levels of *BR-C* and *HR3* transcripts. In other tissues, *E75*, *E74*, *BR-C* and *FTZ-F1* (but not *HR3* and *HR4*) have high levels of expression in the CNS and hindgut. *E75* and *E74* are also highly expressed in the fat body. There is a lower expression for *HR3*, *HR4*, and *FTZ-F1* in the anterior midgut and Malpighian tubules (Fig 2A–2F).

### The effects of a blood meal on transcript expression levels of the ecdysone response genes in the ovary

**Fig 3:**
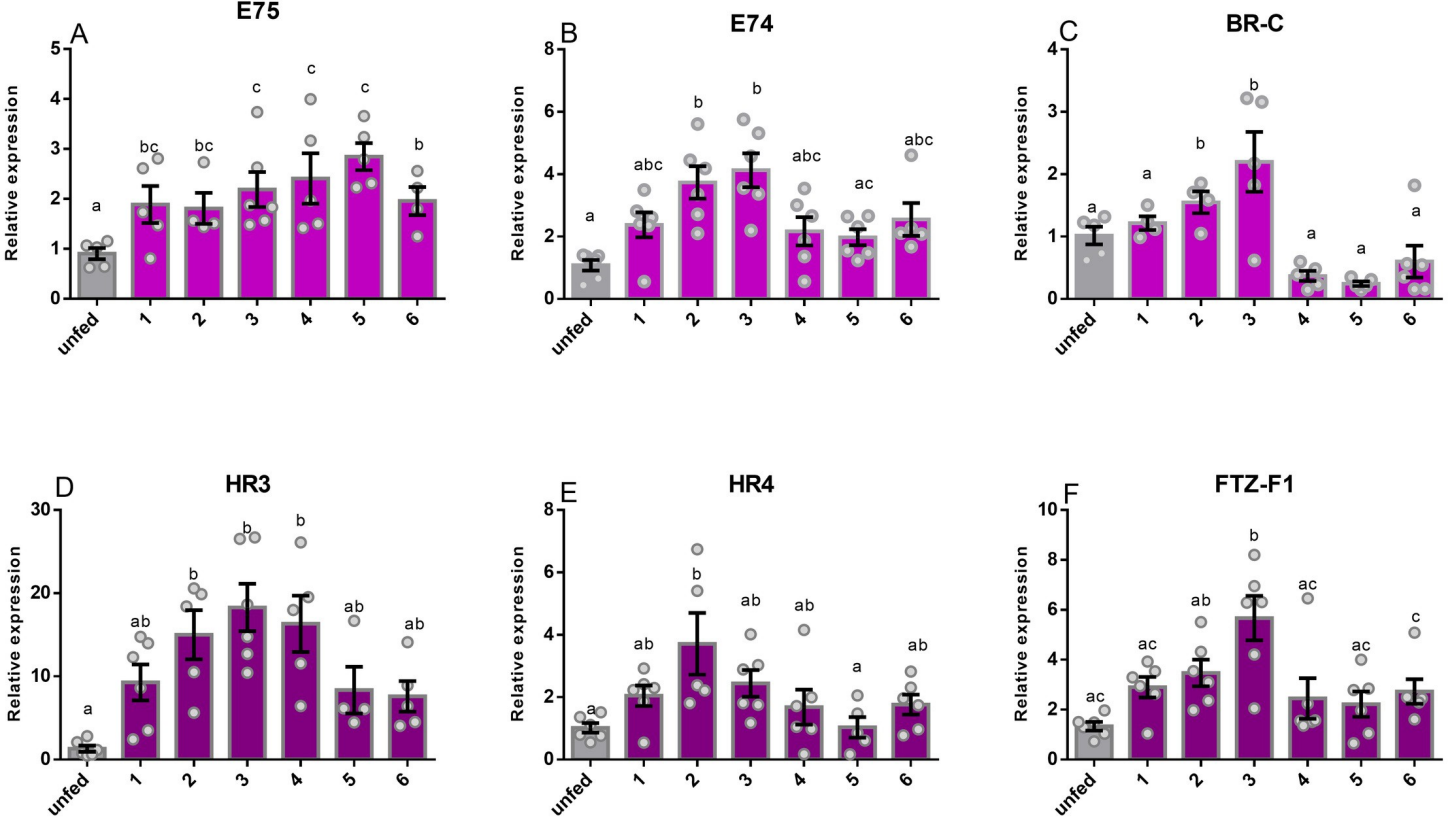
Temporal transcript expression of ecdysone response genes in ovaries. Two ovaries were pooled from adult female insects and analyzed from unfed and at 1, 2, 3, 4, 5, and 6 days post blood meal (PBM) and transcript levels were quantified by RT-qPCR. The expression of ecdysone response genes including *E75* (**A**), *E74* (**B**), *BR-C* (**C**), *HR3* (**D**), *HR4* (**E**), and *FTZ-F1*(**F**) were measured. The y axis represents the fold change in expression relative to unfed (value ~ 1) obtained via geometric averaging using *18S rRNA* and *β-actin* as reference genes. The transcript levels were quantified using RT-qPCR and analyzed using the 2^−ΔΔCt^ method. The results are shown as the mean ± SEM (n = 4–6). Statistical analysis was performed with one-way ANOVA and Tukey’s test for post hoc analysis. Means with significant differences (P<0.05) from others are denoted by different letters.

The transcript expression of ecdysone response genes was quantified in the ovaries of unfed adult females at 10 d PE, and then at days PBM (Fig 3A–3F). All ecdysone response genes are elevated in the first few days following a blood meal. *E75* transcript levels are elevated between days 1 and 6 PBM, while *E74*, *BR-C* and *FTZ-F1* levels are elevated in the second and third days PBM, but then decrease over the following days. There is an elevation in *HR3* on day 2, 3 and 4 PBM, followed by a decrease on days 5–6 PBM. *HR4* transcript levels are elevated by day 2 PBM, then decrease on the following days PBM (Fig 3A–3F).

### The effects of knockdown of *E75*, *E74* or *FTZ-F1* transcripts on transcript expression of the Halloween genes, ecdysone receptor, and ecdysone response genes in the ovary, and on hemolymph ecdysteroid titer

**Fig 4:**
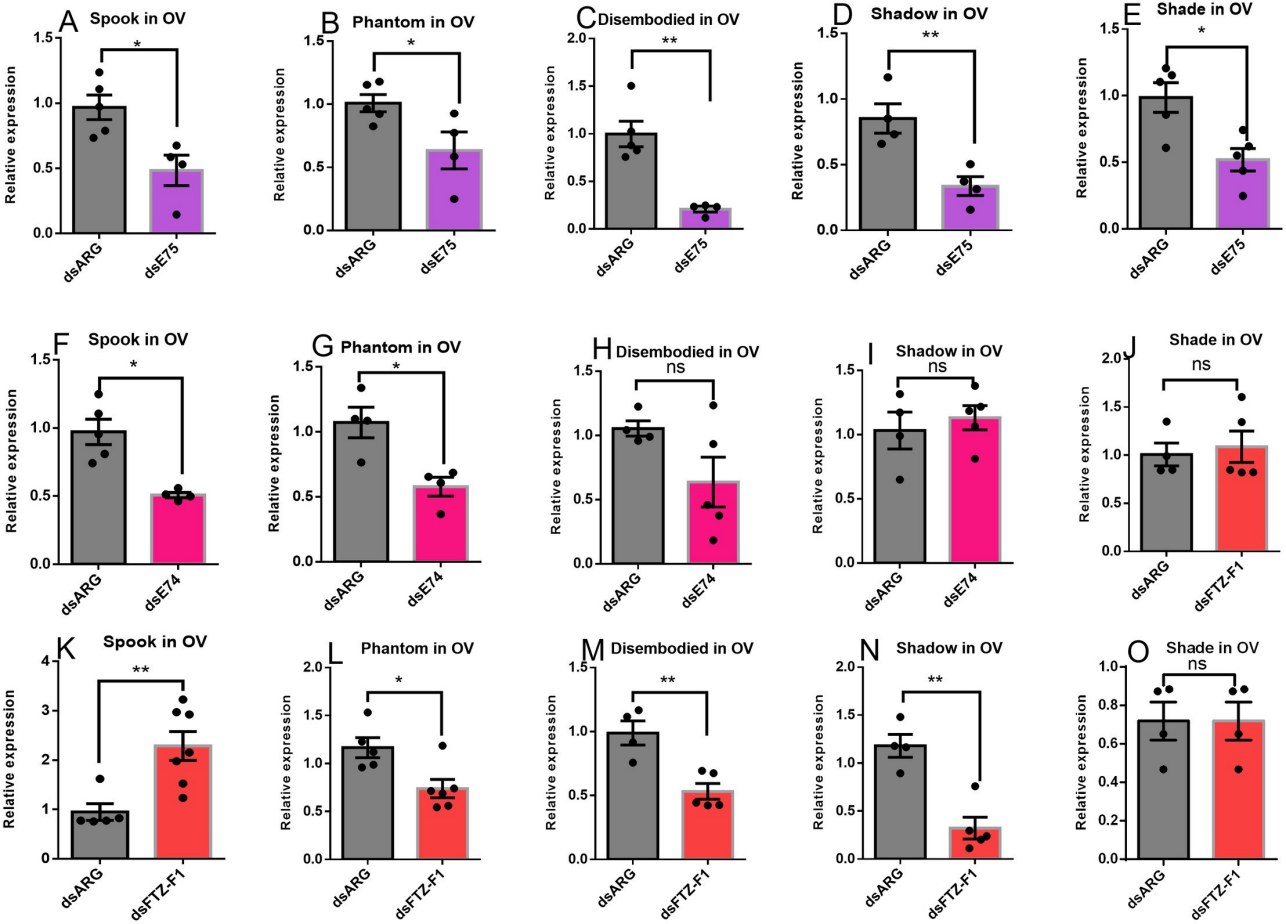
Effects of knockdown of *E75*, *E74* or *FTZ-F1* on transcript expression of Halloween genes (*spook*, *phantom*, *disembodied*, *shadow* and *shade*) in the ovary of adult females 4 days post blood meal. Females were injected with dsRNA and blood fed as described in Materials and Methods. Relative transcript levels were measured using RT-qPCR and analyzed using the 2^−ΔΔCt^ method. Data are means ± SEM (n = 4–7). *Rp49* and *β-actin* were used as reference genes. Statistical analysis was performed by Student’s t‐test. *p < 0.05, **p < 0.01, ns = not significant.

In response to the knockdown of *E75*, the expression of all Halloween genes (*spook*, *phantom*, *disembodied*, *shadow*, and *shade*) in the ovary decreases (Fig 4A–4E). While knockdown of *E74* downregulates transcripts for *spook* and *phantom* in the ovary, the transcripts for *shadow*, *disembodied*, and *shade* are not affected (Fig 4F–4J). dsFTZ-F1 injection downregulates *phantom*, *disembodied*, and *shadow* transcripts in the ovary, increases *spook* expression, but does not affect *shade* (Fig 4K–4O).

**Fig 5:**
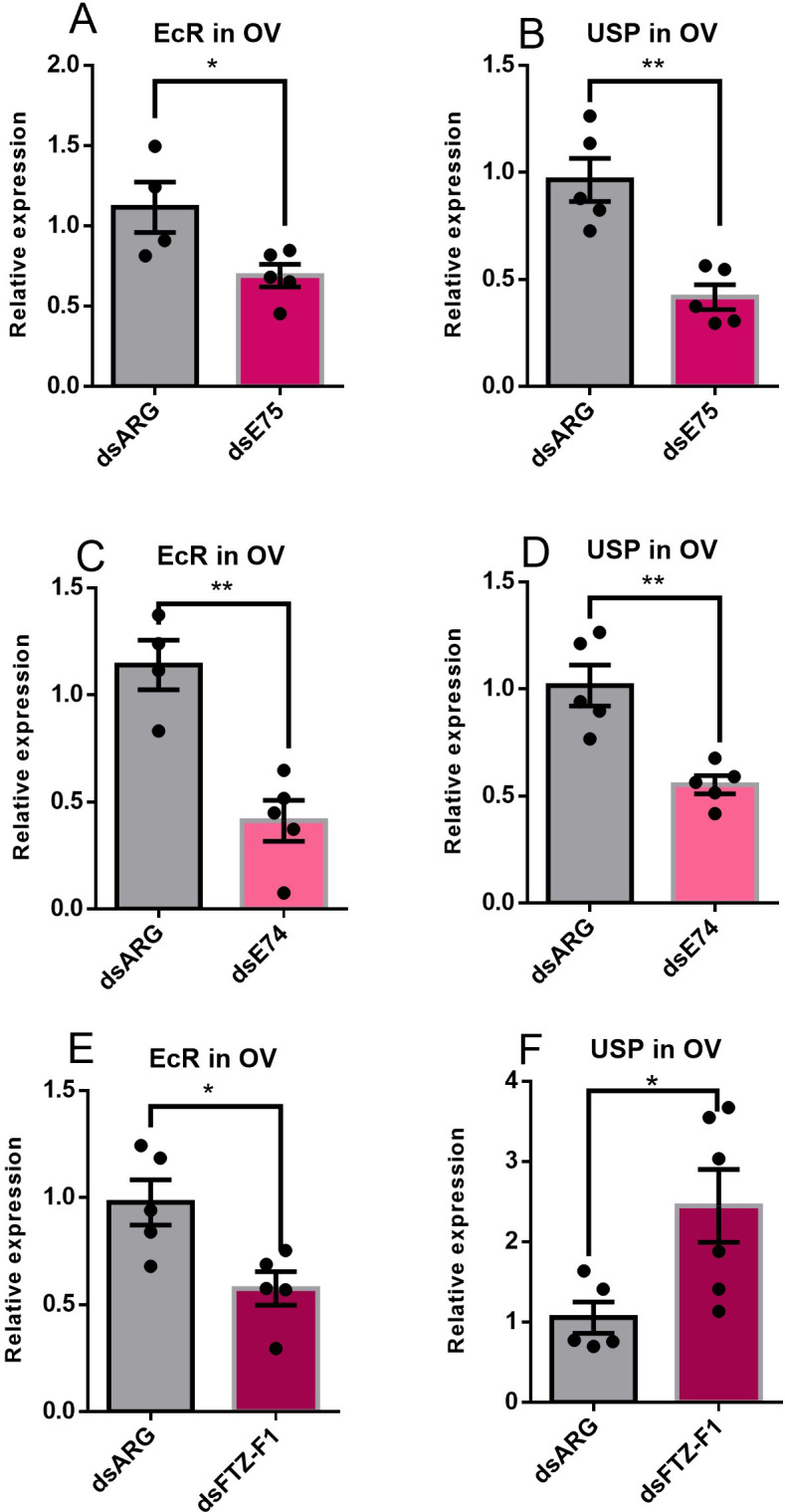
Effects of knockdown of *E75*, *E74* and *FTZ-F1* on expression of the ecdysone receptor in the ovary of adult females 4 days post blood meal. Adult females were injected with dsRNA and fed on a blood meal as described in Materials and Methods. Relative transcript levels were measured using RT-qPCR and analyzed using the 2^−ΔΔCt^ method. Data are means ± SEM (n = 4–6). *Rp49* and *β-actin* were used as reference genes. Statistical analysis was performed by Student’s t‐test. *p < 0.05, **p < 0.01.

Knockdown of *E75* or *E74* expression downregulates the expression of the *EcR* and *USP* transcript expression in the ovary; however knockdown of *FTZ-F1* downregulates *EcR* expression but upregulates *USP* (Fig 5A–5F).

**Fig 6:**
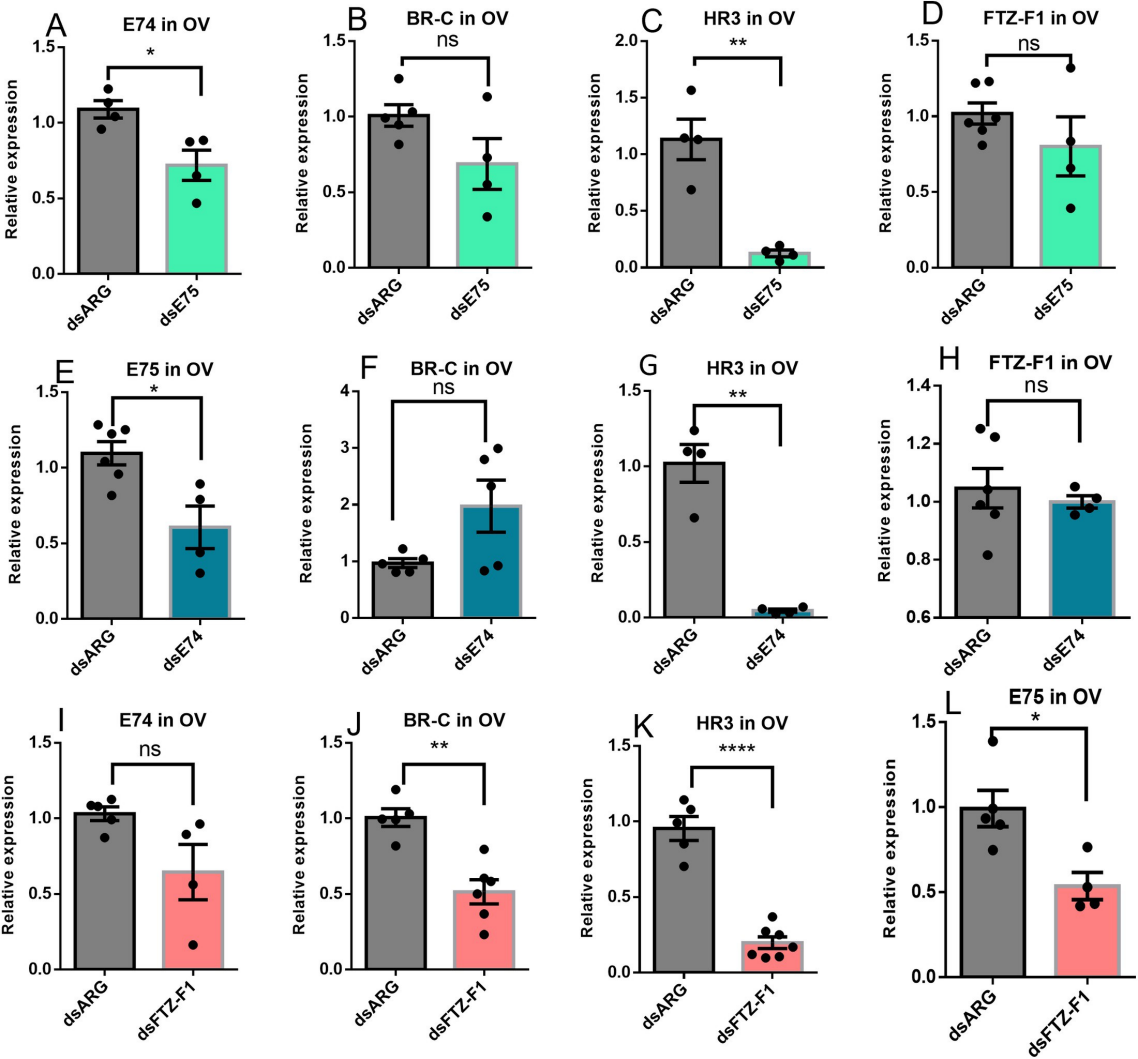
Effects of knockdown of *E75*, *E74* or *FTZ-F1* on transcript expression of ecdysone response genes in the ovary of adult females 4 days post blood meal. Females were injected with dsRNA and blood fed as described in Materials and Methods. Relative transcript levels were measured using RT-qPCR and analyzed using the 2^−ΔΔCt^ method. Data are means ± SEM (n = 4–7). *Rp49* and *β-actin* were used as reference genes. Statistical analysis was performed by Student’s t‐test. *p < 0.05, **p < 0.01, ****p<0.0001, ns = not significant.

The knockdown of the ecdysone response genes, *E75*, *E74*, or *FTZ-F1*, alters expression of other ecdysone response genes with dsE75 significantly reducing expression of *E74* and *HR3* in the ovary, but not the transcripts for *BR-C* and *FTZ-F1* (Fig 6A–6D). Similarly, dsE74 reduces the level of expression of *E75* and *HR3* transcripts in the ovary but does not alter expression of *BR-C* or *FTZ-F1* (Fig 6E–6H). Knockdown with dsFTZ-F1 results in a downregulation of the expression of *E75*, *BR-C* and *HR3* but does not alter *E74* expression in the ovary (Fig 6I–6L).

**Fig 7:**
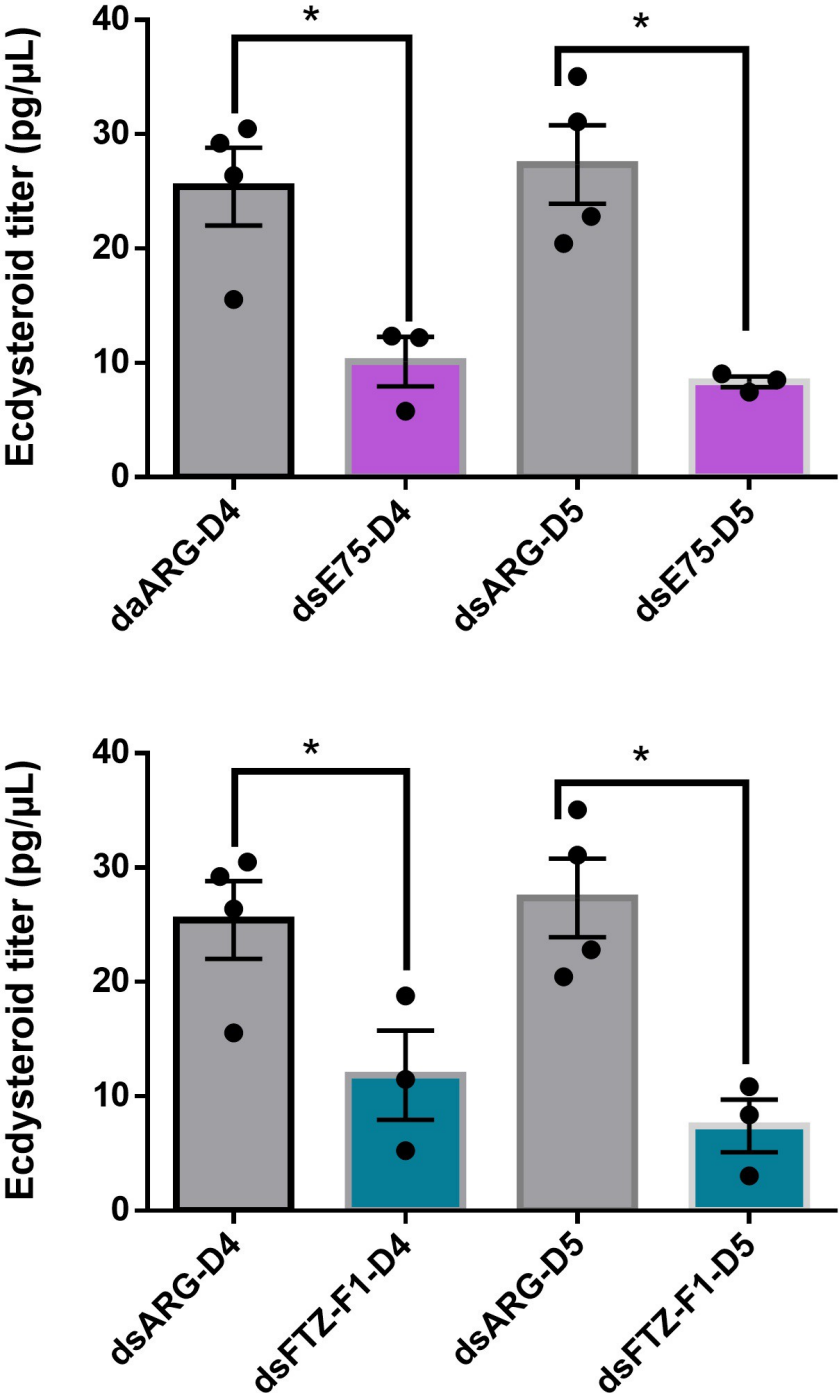
Effect of knockdown of *FTZ-F1* and *E75* on the hemolymph ecdysteroid titer in adult females at days 4 and 5 post blood meal. Bars represent mean ± SEM (n = 3–4). The transcript levels were quantified using RT-qPCR and analyzed using the 2^−ΔΔCt^ method. Data are means ± SEM (n = 3–4). *Rp49* and *β-actin* were used as reference genes. Statistical analysis was performed by Student’s t‐test. *p < 0.05.

Knockdown of transcripts for *E75*and *FTZ-F1* reduces hemolymph ecdysteroid levels significantly compared with control insects on days 4 and 5 PBM (Fig 7A and 7B).

### The effects of knockdown of *E75*, *E74* or *FTZ-F1* transcripts on the transcript expression of the ecdysone receptor, Halloween genes and ecdysone response genes in the fat body

Knockdown of *E75*, *E74* or *FTZ-F1* downregulates the transcripts in the fat body for *EcR* and *USP* at day 4 PBM (S2 Fig). In addition, knockdown of *E75*, *E74* or *FTZ-F1* transcript expression results in a down regulation of all Halloween gene transcripts in the fat body at day 4 PBM (*spook*, *phantom*, *disembodied*, *shadow*, and *shade*) (S3 Fig). Moreover, dsE75, dsE74 or dsFTZ-F1 reduces significantly the expression of the ecdysone response genes in the fat body, except that dsE75 does not alter transcript expression of *FTZ-F1* (S4 Fig).

### Effects of knockdown of ecdysone response genes on egg production in adult females

**Fig 8:**
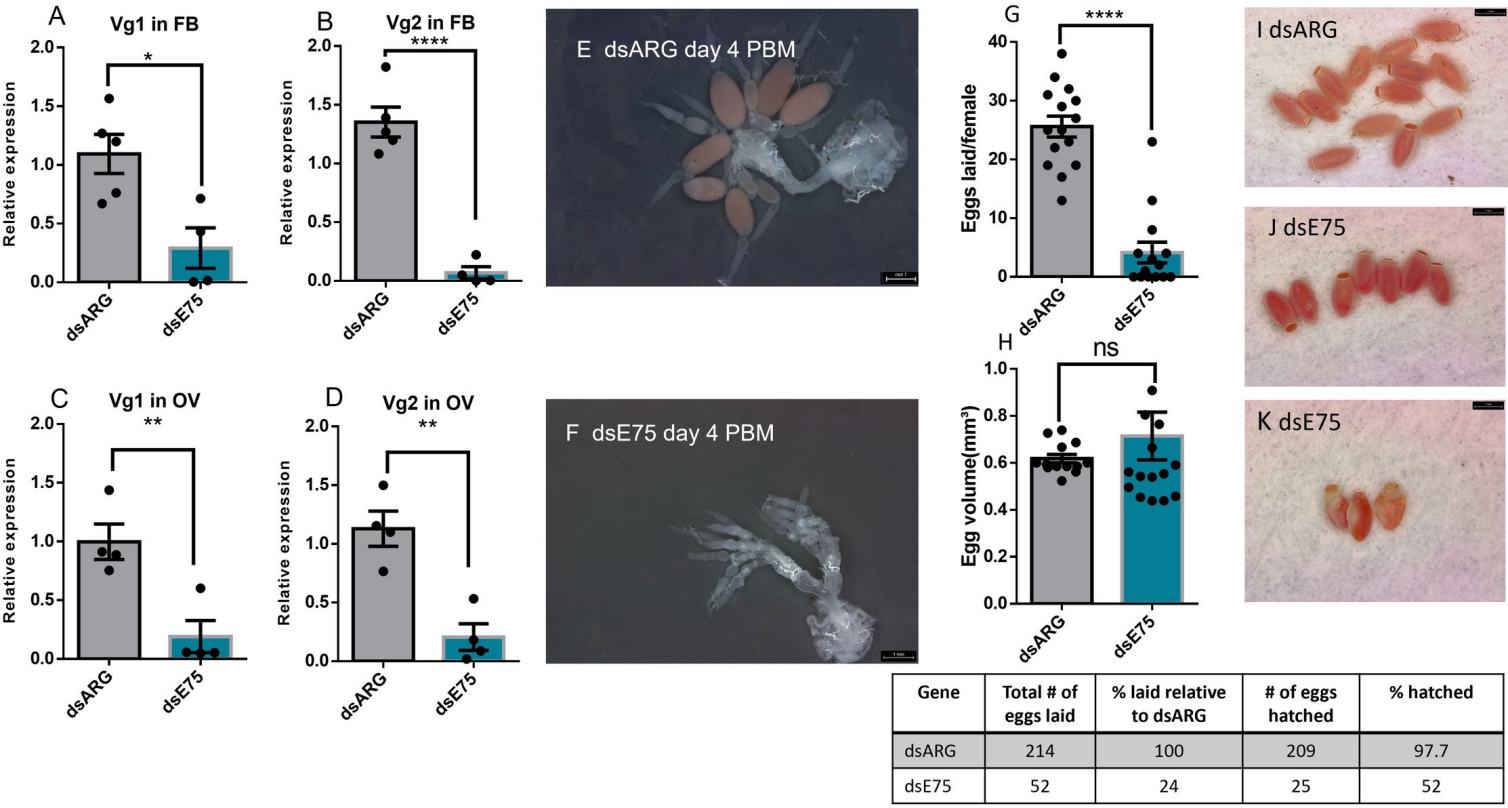
Effect of knockdown of *E75* mRNA in adult females on egg production. RT-qPCR was used to quantify the relative transcript levels of *Vg1* and *Vg2* in the fat body (FB) and the ovary (OV) at 4 d post blood meal (PBM) in dsARG (control) and dsE75-injected females. *Vg1* in FB (A), *Vg2* in FB (B), *Vg1* in ovary (C), and *Vg2* in ovary (D). dsE75-injection affects ovarian phenotype in comparison to dsARG-injected controls (E and F) (n = 4). Eggs laid per female over 15 days PBM (G) (n = 15) and egg volume (H) (n = 15). Phenotypes of laid eggs from insects injected with dsARG (I) or injected with dsE75 (J and K) (n = 15). At 4 days PBM, ovaries were dissected, and images were taken. *Rp49* and *β-actin* were used as reference genes. The transcript levels were quantified using RT-qPCR and analyzed using the 2^−ΔΔCt^ method. Results are presented as mean ± SEM. Student’s t-test was used for statistical analysis. *p < 0.05; **p < 0.01, ****p < 0. 0001, ns = not significant.

**Fig 9:**
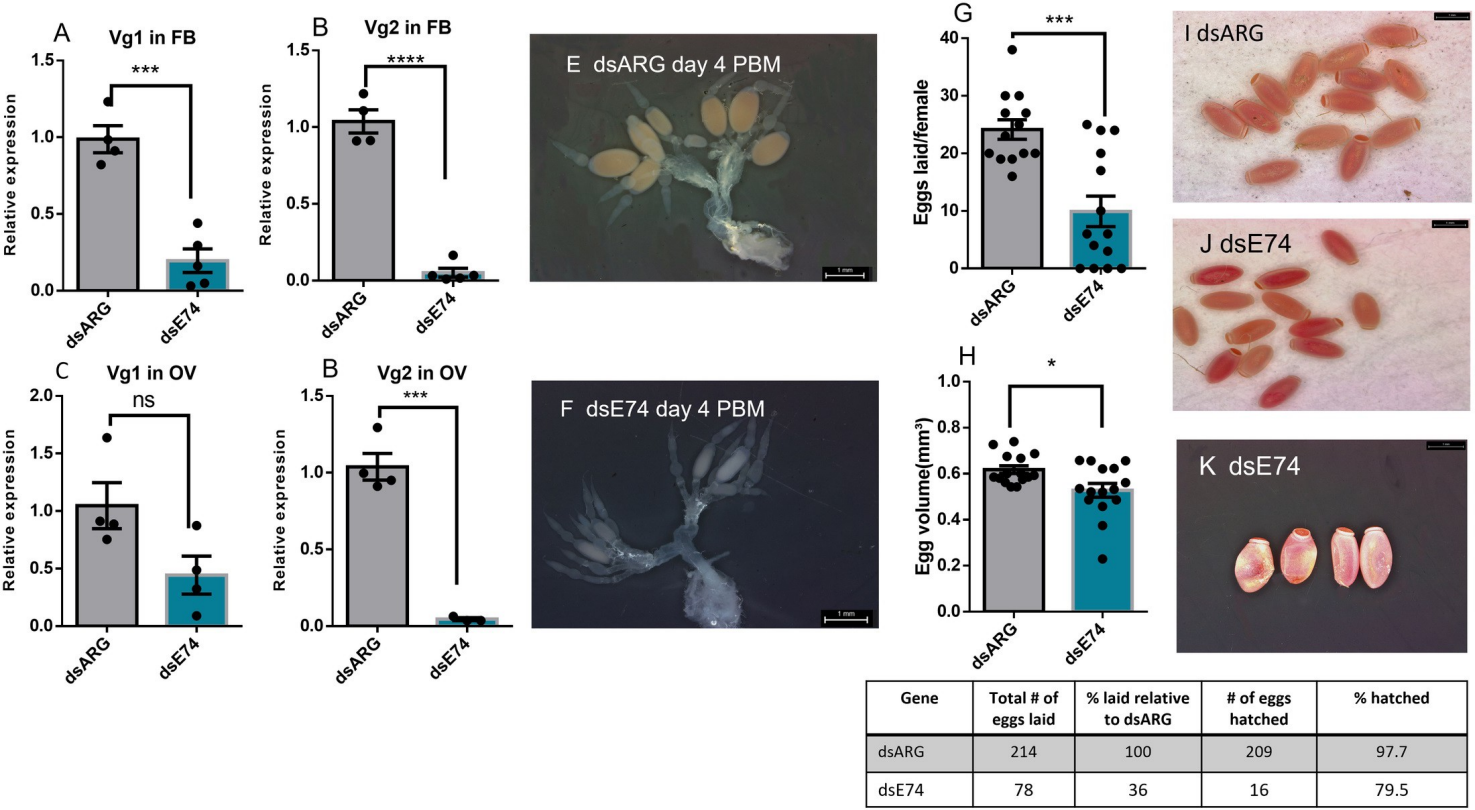
Effect knockdown of *E74* mRNA in adult females on egg production. RT-qPCR was used to quantify the relative transcript levels of *Vg1* and *Vg2* in fat body (FB) and ovary (OV) at 4 d post blood meal (PBM) in dsARG (control) and dsE75-injected females. *Vg1* in FB (A), *Vg2* in FB (B), *Vg1* in ovary (C), and *Vg2* in ovary (D). dsE74-injection affects ovarian phenotype in comparison to dsARG-injected insects (control) (E and F) (n = 4). Eggs laid per female over 15 days PBM (G) (n = 15) and egg volume (H) (n = 15). Phenotypes of laid eggs from insects injected with dsARG (I) or dsE75 (J-K) (n = 15). At 4 days PBM, ovaries were dissected, and images were taken. *Rp49* and *β-actin* were used as reference genes. The transcript levels were quantified using RT-qPCR and analyzed using the 2^−ΔΔCt^ method. Results are presented as mean ± SEM. Student’s t-test was used for statistical analysis. ***p < 0.001, ****p < 0. 0001.

**Fig 10:**
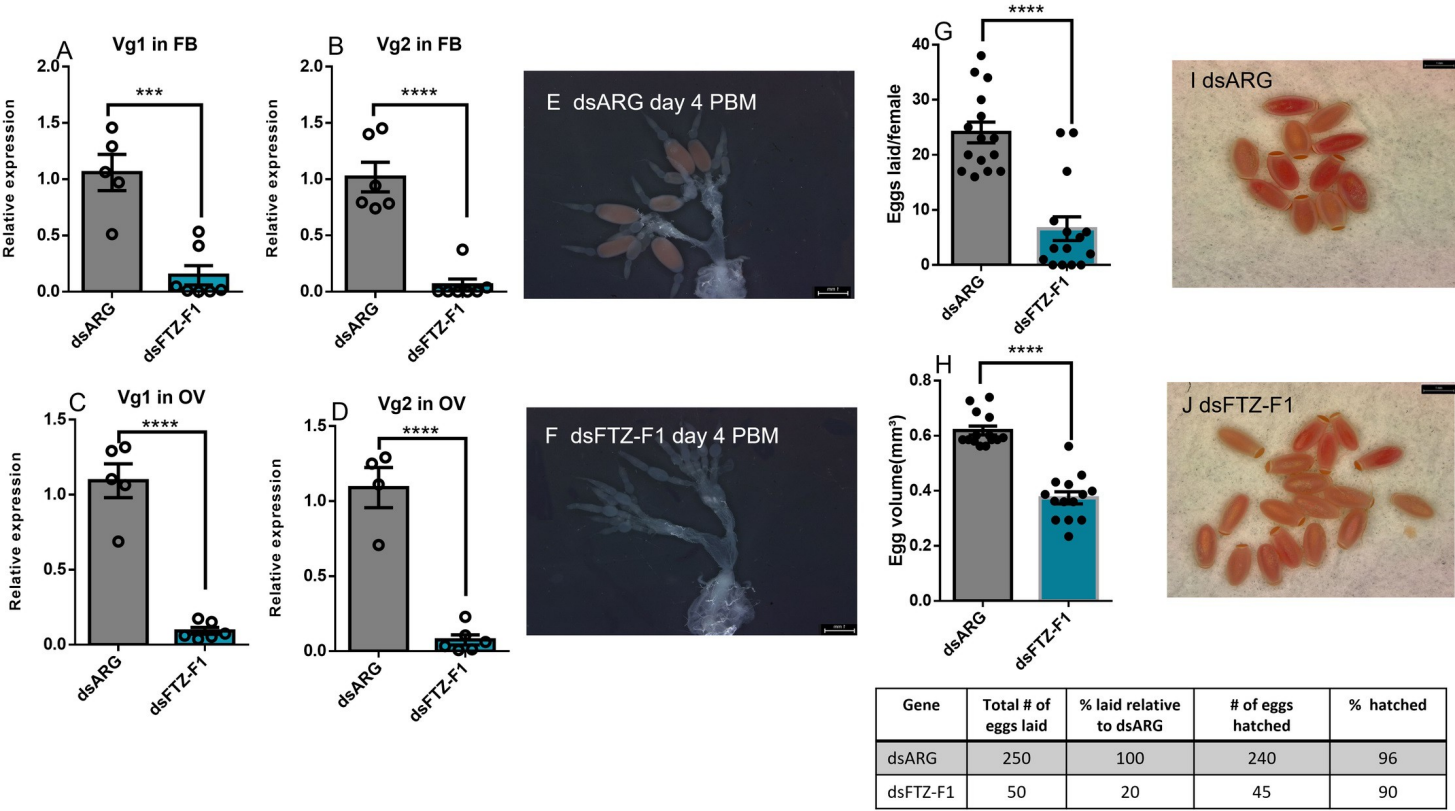
Effect knockdown of *FTZ-F1* mRNA in adult females on egg production. RT-qPCR was used to quantify the relative transcript levels of *Vg1* and *Vg2* in fat body and ovary at 4 d post blood meal in dsARG (control) and dsFTZ-F1-injected females. *Vg1* in FB (A), *Vg2* in FB (B), *Vg1* in ovary (C), and *Vg2* in ovary (D). dsFTZ-F1-injection affects ovarian phenotype in comparison to dsARG-injected insects (control) (E and F) (n = 4). Eggs laid per female over 15 days PBM (G) (n = 15) and egg volume (H) (n = 15). Phenotypes of laid eggs from insects injected with dsARG (I) and insects injected with dsFTZ-F1 (J-K) (n = 15). At 4 days PBM, ovaries were dissected, and images were taken. *Rp49* and *β-actin* were used as reference genes. The transcript levels were quantified using RT-qPCR and analyzed using the 2^−ΔΔCt^ method. Results are presented as mean ± SEM. Student’s t-test was used for statistical analysis. ***p < 0.001, ****p < 0. 0001.

Unfed adult females 7 d PE were injected with dsRNA and then blood fed and mated on 10 d PE. The efficacy of dsRNA treatment was monitored in the ovaries and fat body 4 d PBM (during vitellogenesis) and *E75*, *E74*, and *FTZ-F1* transcript levels found to be significantly reduced in knockdown insects (S1 Fig). At 4 d PBM, *Vg1* and *Vg2* transcript expression levels are significantly lower in the fat body and ovaries following dsE75, dsE74, or dsFTZ-F1-injection as compared to dsARG-injection (Figs 8A–8D, 9A-9D and 10A-10D). At day 4 PBM, the ovaries of female *R*. *prolixus* injected with dsRNA were dissected and photographed. *E75*, *E74*, or *FTZ-F1* knockdown significantly reduces oogenesis, with ovaries producing fewer mature oocytes when compared to dsARG-injected insects. The number of growing follicles (vitellogenic follicles) is also significantly reduced in dsE75, dsE74, or dsFTZ-F1-injected insects compared to dsARG treatment (Figs 8E, 8F, 9E, 9F, 10E and 10F). Knockdown of *E75*, *E74*, or *FTZ-F1* also dramatically decreases the number of eggs laid per female (to 24%, 36%, and 20%, respectively) compared to controls and decreases the hatching rate (to 52%, 79.5%, 90%, respectively) of those few eggs laid (Figs 8G, 9G and 10G). The egg volume of those eggs laid from dsE74 or dsFTZ-F1-injected insects is smaller, compared to those from controls, while dsE75 injection does not alter egg volume (Figs 8J, 9J and 10J). Moreover, both dsE75 and dsE74-injected insects produce some eggs that have an abnormal egg shape (lateral compression) (Figs 8K and 9K) compared with the normal shape (Figs 8J and 9J). The eggs produced by dsFTZ-F1-injected insects appear normal in shape (Fig 10J).

**Fig 11:**
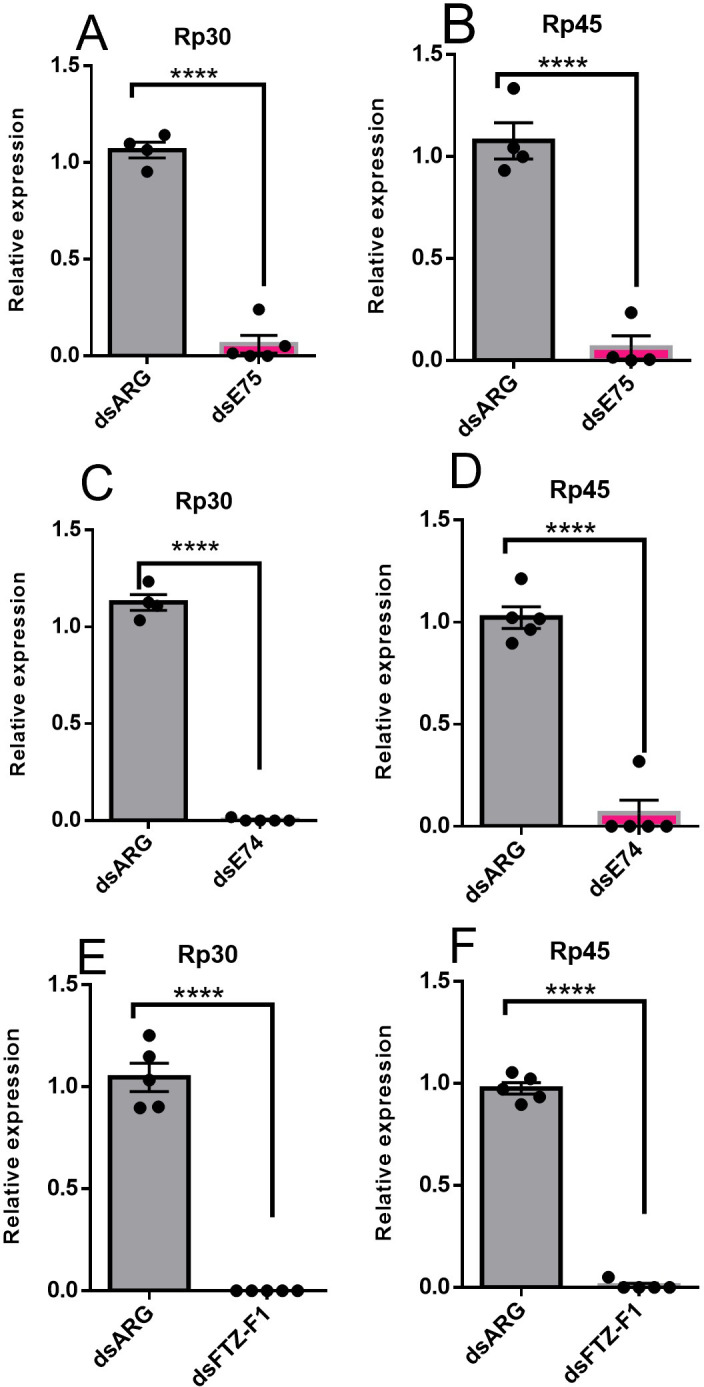
The effect of RNAi-mediated knockdown of *E75*, *E74* or *FTZ-F1* transcripts on expression of transcripts involved in choriogenesis (*Rp30* and *Rp45*) in the ovary 4 days post blood meal (PBM). Relative transcript levels of *Rp30* and *Rp45* were measured using RT-qPCR and analyzed using the 2^−ΔΔCt^ method. The results are shown as mean ± SEM (n = 4–5). *Rp49* and *β-actin* were used as reference genes. Statistical analysis was performed using Student’s t‐test. ****p < 0.0001.

The effect of knockdown of *E75*, *E74*, or *FTZ-F1* on choriogenesis was studied by quantifying the transcript expression for the choriogenesis genes, *Rp30* and *Rp45*, in ovaries on day 4 PBM. Interestingly, *Rp30* and *Rp45* transcripts are significantly reduced in insects injected with dsE75, dsE74, or dsFTZ-F1 (Fig 11).

## Discussion

Insect development, molting, and metamorphosis are dependent on 20E. Cytochrome P450 monooxygenases, encoded by Halloween genes *spook* (*spo*), *phantom* (*phm*), *disembodied* (*dib*), *shadow* (*sad*), and *shade* (*shd*), are involved in ecdysteroid biosynthesis. The overall ecdysteroid signal is regulated by a large number of transcription factors (ecdysone response genes) [8]. In terms of ecdysteroid biosynthesis, transcription factors have mainly been studied in relation to their function in prothoracic glands (PGs) in non-adult insects. However, ecdysteroid biosynthesis occurs in adult stages, in the absence of PGs, with the ovaries as the site of biosynthesis [6, 52]. Furthermore, and importantly, these transcription factors are highly expressed in non-ecdysteroidogenic tissues since they participate in other ecdysteroid-mediated effects. Our previous study illustrated the importance of ecdysteroid for egg production in *R*. *prolixus*, and knocking down the ecdysone receptor and *shade* reduces the number of eggs made and laid, and influences the maturation of oocytes, suggesting ecdysteroid influences vitellogenesis. Furthermore, the abnormal shape of the few eggs laid by insects injected with dsEcR, dsUSP, or dsshade, as well as the decrease in hatching of those eggs, suggests that the eggs are not capable of successful choriogenesis. This was supported by the significant reduction in expression of the chorion gene transcripts (*Rp30* and *Rp45*) in insects injected with dsEcR, dsUSP, or dsshade [37]. In the current study, we have investigated the role of the ecdysone response genes in the regulation of egg production by first identifying them in an ovary transcriptome, examining their tissue distribution, and then examining the effects of knockdown of their transcript expression. Here we find that the ecdysone response genes participate in the 20E regulatory hierarchy controlling vitellogenesis and chorion formation in female *R*. *prolixus*, and knockdown of their transcripts disrupts egg production. We identified *E74*, *E75*, *HR3*, *HR4*, and *FTZ-F1* with significant expression in the ovary, fat body, and brain, which is consistent with reports of other insects [8, 28, 53–57]. The expression of ecdysone response genes in the ovary increases following a blood meal in *R*. *prolixus*, and likely mediates feedback to increase ecdysteroid biosynthesis via the Halloween genes, and thereby increase hemolymph ecdysteroid titer as is necessary during vitellogenesis for egg production. Knockdown of the ecdysone response genes in *R*. *prolixus* by RNAi significantly decreases *Vg1* and *Vg2* expression in the fat body and ovaries, reduces the titer of ecdysteroid in the hemolymph, and interferes with normal egg production. Further evidence of feedback is shown by knockdown of *E75*, *E74* or *FTZ-F1*, which downregulates the transcripts in the fat body and ovary for *EcR* and *USP* at day 4 PBM. In addition, knockdown of *E75*, *E74* or *FTZ-F1* expression results in a downregulation of all Halloween gene transcripts at day 4 PBM *(spook*, *phantom*, *disembodied*, *shadow*, and *shade*). Moreover, dsE75, dsE74, or dsFTZ-F1 mostly reduces the expression of ecdysone response genes in the ovary and fat body. These data suggest ecdysone response genes increase the expression of steroidogenic enzymes in the ovary and fat body via a positive feedback loop, resulting in dramatic increases in ecdysteroid levels in the hemolymph. This data confirms the complex interaction between ecdysteroid biosynthesis, its receptor and the ecdysone response genes.

Ecdysone response genes have previously been reported to be induced by ecdysteroids and are implicated in the regulation of vitellogenesis and female reproduction in many insects [7, 56, 58]. In *A*. *aegypti* the blood meal triggers the 20E cascade with a high expression of genes in the ovary and fat body leading to activation of yolk protein precursor genes in the fat body [30, 59]. Moreover, in the fat body, EcREs in the 5-upstream region of the *Vg* gene have been identified, along with those of *E74*, *E75*, and *BR-C*, indicating direct and indirect regulation by 20E [30, 31, 60]. In addition, in *A*. *aegypti*, a blood meal also activates three early *E75* isoforms and elevates their expression in the fat body during vitellogenesis, with two peaks appearing at 4 h and 24 h PBM [59]. Moreover, a blood meal induces an upregulation of *AaE74* transcript levels in the fat body and ovary, with the highest levels occurring during the peak of vitellogenesis [61]. Our data supports the presence of the ecdysteroidogenic transcription factors in the fat body and ovary and also their upregulation following a blood meal.

Disruption of *FTZ-F1* expression in *D*. *melanogaster* and *T*. *castaneum* causes abnormal follicle development, a decrease in the synthesis of Vg, blocked maturation of the oocyte and ovulation failure [24, 62]. In addition, in *N*. *lugens*, *Vg* expression is significantly reduced 24 hours following injection of dsE74A and the decline is more pronounced 48 hours after injection [63]. Knockdown of *E75*, *E74*, or *FTZ-F1* in *R*. *prolixus* decreases egg production, resulting in fewer eggs being laid than controls, and a reduced hatching rate, especially for knockdown of the transcript for *E75*. A small egg size was found from the dsE74 and dsFTZ-F1-injected insects, but interestingly, this did not appear to affect the hatching rate of eggs laid by dsFTZ-F1-injected insects. In part these results could be caused by lower ecdysteroid titer in the hemolymph coupled with lower expression of vitellogenin genes, influencing the maturation of oocytes and their growth. We have previously reported that in *R*. *prolixus*, disruption of ecdysteroid signaling, via knockdown of *ECR*, *USP* and Halloween genes, affects vitellogenin expression, chorion gene expression, oocyte maturation, and egg-laying [37], and the present data illustrates that the effects of ecdysteroids are mediated by the ecdysone response genes. In *T*. *castaneum*, egg-laying is completely blocked with the knockdown of *E75*, *HR3*, *EcR*, *USP*, *SVP*, *FTZ-F1* and *HR4* [24]. Moreover, knockdown of *ECR* and *BR-C* in *Cimex lectularius* results in a reduction in the number of eggs laid [64]. Also, in *N*. *lugens*, silencing *E93*, one of the early ecdysone response genes, suppresses ovary development, resulting in fewer eggs produced [65]. In addition to playing a role in egg production, ecdysteroid signaling is involved in choriogenesis in adult female desert locusts through the nuclear receptor complex [65, 66]. The ecdysone receptor and *shade* play important roles in the regulation of vitellogenesis in *R*. *prolixus* [37]. 20E induces Vg synthesis in the fat body, but how it regulates vitellogenesis is unclear. Currently, we do not know whether 20E influences oocyte maturation directly, or whether the signals released by maturing oocytes influence vitellogenesis. This study investigated the role of genes downstream of 20E signaling on egg production and transcription of vitellogenin. As a result, RNAi knockdown of genes that encode proteins involved in ecdysteroid signaling result in severe defects in the maturation of the oocytes and egg production. Our findings are consistent with reports showing that the downregulation of *E75*, *E74*, *HR3*, and *FTZ-F1* affects the expression of Halloween genes and other transcription factors in the ovary and fat body. Several transcription factors have been identified as responsible for Halloween gene expression. In the PGs, *E75*, *FTZ-F1*, and *HR3*, which are downstream of 20E-EcR signaling, regulate ecdysteroid biosynthesis [8, 67]. In *D*. *melanogaster*, knockdown of *BR-C* and *HR3* in the ring gland leads to a reduced expression of ecdysone biosynthesis related genes (*phantom*, *disembodied* and *shadow*) while downregulation of *E75* reduces the expression of *phantom* [7, 8, 11, 67–70]. In *R*. *prolixus* females *E75* expression is also influenced by dsFTZ-F1and dsE74, whereas *HR3* expression is influenced by dsE75, dsE74 and dsFTZ-F1. Similar feedback of the ecdysone response genes on genes responsible for ecdysteroid biosynthesis or the 20E-EcR pathway have been shown in other studies. For example, the levels of mRNA of *EcR* and *USP* are decreased by 80–90% in *B*. *mori E93* RNAi larvae compared to their control counterparts [71]. Furthermore, in *B*. *mori* E75 RNAi disrupts the 20E-triggered transcriptional cascade by lowering EcR-B1 and USP protein levels [72] and in *D*. *melanogaster* early L3 larvae, RNAi of *FTZ-F1* decreases *EcR* expression, indicating that *FTZ-F1* is necessary to maintain *EcR* expression in this stage [69].

This study helps us understand how ecdysone response genes regulate *Vg* expression in *R*. *prolixus* and the feedback between ecdysteroid and ecdysone response genes. However, how these transcription factors regulate Halloween gene transcription still needs further investigation.

## Supporting information

S1 Fig Efficiency of knockdown of *E75*, *E74* and *FTZ-F1* transcripts in the ovary (OV) and fat body (FB) 4 days post blood meal in adult female *R*. *prolixus*. Females were injected with dsRNA as described in Materials and Methods. Relative levels of the transcripts were measured in the fat body and ovaries using RT-qPCR. Data indicate means ± SEM (n = 4–6). **p < 0. 01; ***p<0.001; ****p < 0. 0001, Statistics were performed using Student’s t‐test.
(DOCX)

S2 Fig Effects of knockdown of *E75*, *E74* or *FTZ-F1* on transcript expression of *EcR* and *USP* in the fat body (FB) of *R*. *prolixus* adult females. Knockdown of these genes downregulates the transcript levels of the ecdysone receptor, in the fat body at 4 days post blood meal of adult female *R*. *prolixus*. Females were injected as described in Materials and Methods. Relative transcript levels were measured using RT-qPCR analyzed using the 2^−ΔΔCt^ method. *Rp49* and *β-actin* were used as reference genes. Data indicate means ± SEM (n = 4). *p < 0.05, ***p<0.001. Statistical analysis was performed by Student’s t‐test.
(DOCX)

S3 Fig Effects of knockdown of *E75*, *E74* or *FTZ-F1* on transcript expression of Halloween genes in the fat body (FB) of *R*. *prolixus* adult females. Knockdown of these genes in the FB downregulates the transcript levels of the Halloween genes (*spook*, *phantom*, *disembodied*, *shadow*, and *shade*) in the FB at 4 days post blood meal of adult female *R*. *prolixus*. Females were injected as described in Materials and Methods. Relative transcript levels were measured using RT-qPCR and analyzed using the 2^−ΔΔCt^ method. *Rp49* and β-*actin* were used as reference genes. Data indicate means ± SEM (n = 4–6). *p < 0.05, **p < 0.01, ***p < 0. 001; ****p < 0. 0001. Statistical analysis was performed by Student’s t‐test.
(DOCX)

S4 Fig Effects of knockdown of *E75*, *E74* or *FTZ-F1* on transcript expression of ecdysone response genes in the fat body (FB) of *R*. *prolixus* adult females. Knockdown of these genes in the FB downregulates the transcript levels of some of the ecdysone response genes in the fat body at 4 days post blood meal of adult female *R*. *prolixus*. Females were injected as described in Materials and Methods. Relative transcript levels were measured using RT-qPCR and analyzed using the 2^−ΔΔCt^ method. .*Rp49* and *β*-*actin* were used as reference genes. Data indicate means ± SEM (n = 4–6). *p < 0.05, **p < 0.01, ***p<0.001, ****p < 0. 0001; ns = not significant. Statistical analysis was performed by Student’s t‐test.
(DOCX)

S1 Table Gene specific primers for qPCR and RNAi. (DOCX)

S2 Table Ecdysone response genes (*E75*, *E74*, *BR-C*, *HR3*, *HR4* and *FTZ-F1*) sequences from the *R*. *prolixus* genome and ovary transcriptome. (DOCX)
