# Supplementary material for: A critical role for ecdysone response genes in regulating egg production in adult female Rhodnius prolixus
Source: PLoS One. 2023 Mar 20;18(3):e0283286. doi: 10.1371/journal.pone.0283286 (PMC10027210; doi:10.1371/journal.pone.0283286)
Supplement: S1 Fig — Females were injected with dsRNA as described in Materials and Methods. Relative levels of the transcripts were measured in the fat body and ovaries using RT-qPCR. Data indicate means ± SEM (n = 4–6). **p < 0. 01; ***p<0.001; ****p < 0. 0001, Statistics were performed using Student’s t‐test. (DOCX) [file pone.0283286.s001.docx]

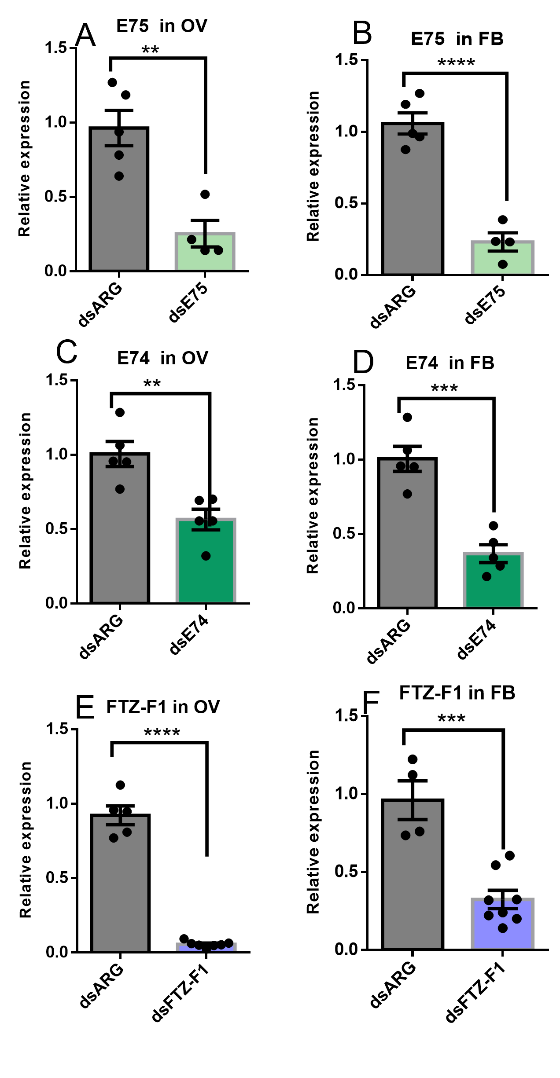


**S1 Fig. Efficiency of knockdown of *E75*, *E74* and *FTZ-F1* transcripts in the ovary (OV) and fat body (FB) 4 days post blood meal in adult female *R. prolixus*.** Females were injected with dsRNA as described in Materials and Methods. Relative levels of the transcripts were measured in the fat body and ovaries using RT-qPCR. Data indicate means ± SEM (n=4-6). **p < 0. 01; ***p<0.001; ****p < 0. 0001, Statistics were performed using Student's t‐test.
