# Supplementary material for: A critical role for ecdysone response genes in regulating egg production in adult female Rhodnius prolixus
Source: PLoS One. 2023 Mar 20;18(3):e0283286. doi: 10.1371/journal.pone.0283286 (PMC10027210; doi:10.1371/journal.pone.0283286)
Supplement: S2 Fig — Knockdown of these genes downregulates the transcript levels of the ecdysone receptor, in the fat body at 4 days post blood meal of adult female R. prolixus. Females were injected as described in Materials and Methods. Relative transcript levels were measured using RT-qPCR analyzed using the 2−ΔΔCt method. Rp49 and β-actin were used as reference genes. Data indicate means ± SEM (n = 4). *p < 0.05, ***p<0.001. Statistical analysis was performed by Student’s t‐test. (DOCX) [file pone.0283286.s002.docx]

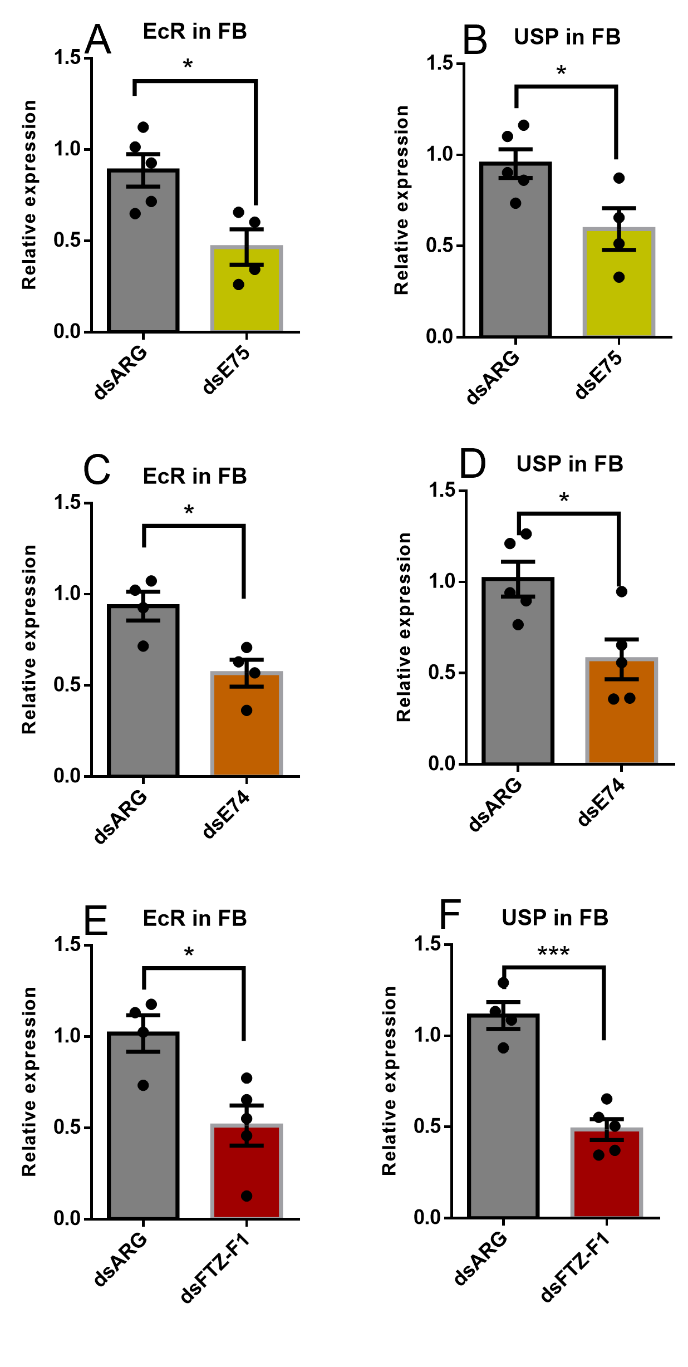


**S2 Fig. Effects of knockdown of *E75, E74* or *FTZ-F1* on transcript expression of *EcR* and *USP* in the fat body (FB) of *R. prolixus* adult females.** Knockdown of these genes downregulates the transcript levels of the ecdysone receptor, in the fat body at 4 days post blood meal of adult female *R. prolixus*. Females were injected as described in Materials and Methods. Relative transcript levels were measured using RT-qPCR. Data indicate means ± SEM (n = 4). *p < 0.05, ***p<0.001. Statistical analysis was performed by Student's t‐test.
