# Supplementary material for: A critical role for ecdysone response genes in regulating egg production in adult female Rhodnius prolixus
Source: PLoS One. 2023 Mar 20;18(3):e0283286. doi: 10.1371/journal.pone.0283286 (PMC10027210; doi:10.1371/journal.pone.0283286)
Supplement: S1 Table — (DOCX) [file pone.0283286.s005.docx]

**S1 Table. Gene specific primers for qPCR and RNAi.**

|  |  | |
| --- | --- | --- |
| VectorBase: RPRC000853 | Rhopr-E75  qPCR  Forward | AGGTTTTCACCAGCCATCAG |
|  | Rhopr-E75  qPCR Reverse | GCCCGCTTTCAAAAGTGTAA |
| VectorBase: PRC007565 | Rhopr-E74 qPCR  Forward | ACCGGGAGAAGGGTGTATTT |
|  | Rhopr-E74  qPCR Reverse | AGAGCACGTCCCATTGTTTC |
| VectorBase: RPRC003967 | Rhopr-BR-C  qPCR  Forward | TCGAGGACCTACATGCTCTTG |
|  | Rhopr-RB-C  qPCR Reverse | TTGTCCAAACCCATGGAGAC |
| VectorBase: RPRC003681 | Rhopr-HR3  qPCR  Forward | GTGTGCGGAGACAAATCAAG |
|  | Rhopr-HR3  qPCR Reverse | CATGCCCAGCCTTAGACATT |
| VectorBase RPRC012796 | Rhopr-HR4  Forward | TGAAAGGACTAGCGGGAGAA |
|  | Rhopr-HR4  qPCR Reverse | CATTGCTAACGTGGCGTCTA |
| VectorBase: RPRC002968 | Rhopr-FTZ-F1  Forward | GGATTAGCATCACCTGGCATA |
|  | Rhopr-FTZ-F1  qPCR Reverse | GCTTTCGCAAGAGAAGATGC |
| VectorBase: RPRC014174 | Rhopr-EcR  Forward | AATGCTGAATACGCCTTGCT |
|  | Rhopr-EcR  qPCR Reverse | ATCGTAGGAGGCCTTGGTTT |
| VectorBase: RPRC013330 | Rhopr-USP  Forward | TCAGTGCAGGTTGGAATGAG |
|  | Rhopr-USP  qPCR Reverse | TCTGATGAGCGTTGTTCCTG |
| VectorBase; RPRC011241 | Rhopr-spook Forward qPCR | TGGCATTCTCCGATTGGTCT |
|  | Rhopr-spook Reverse qPCR | TCATTGAGCAACGTGTCCAGT |
| VectorBase: RPRC009372 | Rhopr-phantom Forward qPCR | TGCCATACACGGAAGCATGT |
|  | Rhopr-phantom Reverse qPCR | CGATAGCCTGCCAGTTCAGT |
| VectorBase: RPRC011595 | Rhopr-disembodied Forward qPCR | TTGCACACTACCGTTTGTCG |
|  | Rhopr- disembodied Reverse qPCR | AATGCGAGCAAGTGGTTTTT |
| VectorBase: RPRC006417 | Rhopr- Shadow Forward qPCR | GAGGCAAGTTTTCGAAGTGG |
|  | Rhopr-Shadow Reverse qPCR | TGATTCATAATTCGGCGATG |
| VectorBase: RPRC006945 | Rhopr-Shade Forward qPCR | ATTCTTTGGGCTCCCATTCT |
|  | Rhopr-Shade Reverse qPCR | GCCATGAACACACTTTGCAC |
| GenBank: EF187283.1 | Rhopr-RP30 Forward qPCR | CCAAACCAGTACCCGTTGTT |
|  | Rhopr-RP30 Reverse qPCR | GGTACATGGGTGGTGTAGGG |
| GenBank: EF187284.1 | Rhopr-RP45 Forward qPCR | GCAATGTTCGTCCCGTAGTT |
|  | Rhopr-RP45  Reverse qPCR | TATTTCTGGGTGACGGGAAC |
| VectorBase: RPRC013511 | Rhopr-Vg1  qPCR Forward | TTGCTAGTCGCATGAACCTG |
|  | Rhopr-Vg1  qPCR Reverse | TTTAGTGGTGCATCGCTCTG |
| VectorBase: RPRC002109 | Rhopr-Vg2 Forward qPCR | TCCATTGCCTAACCTCCTTG |
|  | Rhopr-Vg2 Reverse qPCR | GTAAGGACGATGCGGCTAAC |
| VectorBase: RPRC009875 | β-actin qPCR  Forward | AGAGAAAAGATGACGCAGATAATGT |
|  | β-actin qPCR  Reverse | ATATCCCTAACAATTTCACGTTCG |
| GenBank: AJ421962.1 | 18 S rRNA qPCR  Forward | TGTCGGTGTAACTGGCATGT |
|  | 18 S rRNA qPCR  Reverse | TCGGCCAACAAAAGTACACA |
| VectorBase: RPRC014419 | RP49 qPCR  Forward | GTGAAACTCAGGAGAAATTGGC |
|  | RP49 qPCR  Reverse | AGGACACACCATGCGCTATC |

| dsRNA (5´-3´) - *TAATACGACTCACTATAGGGAGA = T7 RNA polymerase promotor | | |
| --- | --- | --- |
| VectorBase: RPRC000853 | Rhopr-E75  Forward | TTCCAAGTGCCAGGAAAAAG |
|  | T7- Rhopr-  E75 Forward | TAATACGACTCACTATAGGGAGATTCCAAGTGCCAGGAAAAAG |
| VectorBase: RPRC000853 | Rhopr-E75  Reverse | AGTCCAAAACCGGGTATCCT |
|  | T7- Rhopr-  EcR Reverse | TAATACGACTCACTATAGGGAGAAGTCCAAAACCGGGTATCCT |
| VectorBase: PRC007565 | Rhopr-E74  Forward | GACCCCCGCAGAGTTACTTC |
|  | T7- Rhopr-E74  Forward | TAATACGACTCACTATAGGGAGAGACCCCCGCAGAGTTACTTC |
| VectorBase: PRC007565 | Rhopr-E74  Reverse | GCCCCAGTGCAGTCTATTTC |
|  | T7- Rhopr-E74  Reverse | TAATACGACTCACTATAGGGAGAGCCCCAGTGCAGTCTATTTC |
| VectorBase : RPRC002968 | Rhopr-FTZ-F1  Forward | ATGCGGAAGATGAGAAACCA |
|  | T7- Rhopr-  FTZ-F1 Forward | TAATACGACTCACTATAGGGAGAATGCGGAAGATGAGAAACCA |
| VectorBase : RPRC002968 | Rhopr-FTZ-F1  Reverse | GAATGTTGCACCATGTCAGC |
|  | T7- Rhopr-  FTZ-F1 Reverse | TAATACGACTCACTATAGGGAGAGAATGTTGCACCATGTCAGC |
