# Supplementary material for: A critical role for ecdysone response genes in regulating egg production in adult female Rhodnius prolixus
Source: PLoS One. 2023 Mar 20;18(3):e0283286. doi: 10.1371/journal.pone.0283286 (PMC10027210; doi:10.1371/journal.pone.0283286)
Supplement: S2 Table — (DOCX) [file pone.0283286.s006.docx]

**S2 Table. Ecdysone response genes (*E75*, *E74*, *BR-C*,*HR3*,*HR4* and *FTZ-F1*) sequences from the *R. prolixus* genome and ovary transcriptome**

| Gene name | Contig Number | Contig Length (bp) | ORF Length (aa) | Domain | complete gene | Ovary Transcriptome |
| --- | --- | --- | --- | --- | --- | --- |
| E75 | RPRC000853 | 750 | 249 | (LBD) &DBD | √ | √ |
| E74 | RPRC007565 | 927 | 308 | EtS | √ | √ |
| BC-R | RPRC003967 | 1269 | 423 | BTB/POZ | √ | √ |
| HR3 | RPRC003681 | 834 | 257 | LBD &DBD | Fragment | √ |
| HR4 | RPRC012796 | 1698 | 565 | (LBD) &DBD | Fragment | √ |
| FTZ-F1 | RPRC002968 | 1857 | 618 | (LBD) &DBD | √ | √ |

Abbreviation; bp, base pair; aa, amino acid; LBD, ligand-binding domain; DBD, DNA-binding domain
